# Modulation of Host Lipid Pathways by Pathogenic Intracellular Bacteria

**DOI:** 10.3390/pathogens9080614

**Published:** 2020-07-28

**Authors:** Paige E. Allen, Juan J. Martinez

**Affiliations:** Vector Borne Disease Laboratories, Department of Pathobiological Sciences, LSU School of Veterinary Medicine, Baton Rouge, LA 70803, USA; pallen7@lsu.edu

**Keywords:** intracellular pathogen, lipids, fatty acids, β-oxidation

## Abstract

Lipids are a broad group of molecules required for cell maintenance and homeostasis. Various intracellular pathogens have developed mechanisms of modulating and sequestering host lipid processes for a large array of functions for both bacterial and host cell survival. Among the host cell lipid functions that intracellular bacteria exploit for infection are the modulation of host plasma membrane microdomains (lipid rafts) required for efficient bacterial entry; the recruitment of specific lipids for membrane integrity of intracellular vacuoles; and the utilization of host lipid droplets for the regulation of immune responses and for energy production through fatty acid β-oxidation and oxidative phosphorylation. The majority of published studies on the utilization of these host lipid pathways during infection have focused on intracellular bacterial pathogens that reside within a vacuole during infection and, thus, have vastly different requirements for host lipid metabolites when compared to those intracellular pathogens that are released into the host cytosol upon infection. Here we summarize the mechanisms by which intracellular bacteria sequester host lipid species and compare the modulation of host lipid pathways and metabolites during host cell infection by intracellular pathogens residing in either a vacuole or within the cytosol of infected mammalian cells. This review will also highlight common and unique host pathways necessary for intracellular bacterial growth that could potentially be targeted for therapeutic intervention.

## 1. Introduction

Lipids are broadly described as hydrophobic or amphipathic molecules that are involved in various biological functions including mediating immune signaling, regulating vesicle trafficking, providing energy storage and production, and structural membrane composition [1,2,3,4,5,6,7,8,9,10]. Lipids are a large, diverse group of molecules that provide various specific functions required by the host cell to maintain homeostasis. These lipids can be divided into various classes that are highly organized and provide very specialized functions [5]. Furthermore, because of the essentiality and multifunctional property of host lipids for cell maintenance, intracellular pathogens have adapted mechanisms to employ a number of these lipids throughout infection for both host cell regulation, and pathogen replication and sustainability [11]. 

Intracellular pathogens modify host lipid composition by regulating host cell uptake of exogenous lipids from the environment and modulating the expression of enzymes involved in the host de novo lipid biosynthetic pathways [12,13,14,15,16]. This alteration in host lipid composition can be beneficial to the pathogen by providing a number of different functions including: (i) Employing storage and/or free lipids for energy production. Fatty acids within host lipid storage compartments, known as lipid droplets (LDs), and free fatty acids can be catabolized by various methods, such as lipolysis, lipophagy, and selective breakdown via phospholipases. These processes differ in that lipolysis releases lipids through hydrolysis, lipophagy utilizes host autophagy machinery to breakdown lipids within lipid droplets, and phospholipases selectively catabolize specific species of lipids some of which are associated with LDs. The lipids released can then be used in the β-fatty acid oxidation (FAO) pathway, which enzymatically breaks down fatty acids to acetyl-CoA for use in oxidative phosphorylation (OXPHOS) for energy production [17]. Pathogens are also able to release and sequester metabolites within these catabolic pathways by use of bacterial phospholipases for self-generation of energy. The regulation of host lipid metabolism to promote shifts in energy production pathways has also been implemented in modulating host cell persistence, pathogen sustainability, and anti-inflammatory mechanisms. (ii) Pathogen membrane production. Some intracellular pathogens have been shown to have unique lipids within their membrane structures that are unlike common bacterial lipids and that are unable to be made by the pathogen machinery encoded in the genome. Thus, is it likely that the lipids are acquired from the host and are incorporated into the pathogen membrane. (iii) Invasion of host signaling pathways and the host immune system. Lipid organization within host membranes play an important role in the regulation of signaling pathways by creating “hot spots,” known as lipid rafts, for enabling the association of receptors with external and internal stimuli. These functional, highly organized membrane regions are utilized by various intracellular bacterial pathogens for entry into the host cell [18]. There are also fatty acid molecules released from LDs, such as arachidonic acids, that are precursors of host immune modulators, such as prostaglandins and leukotrienes, and have a key role in the presentation of disease during pathogen infection [19,20,21]. 

Human pathogenic intracellular bacteria have developed mechanisms that initiate the employment of host lipids to cause disease during mammalian infection. Among the functions that require host lipids are bacterial entry into the host cell, formation of an intracellular compartment, metabolic requirements for bacterial survival and replication, and disease initiation and progression. However, because intracellular bacterial pathogens differ in the mechanisms used to colonize cells, it is likely that host lipid manipulation during infection varies between different bacterial species [22]. Specifically, intracellular bacteria can be separated into those that persist in a vacuolar compartment inside the host cell and those that survive and replicate within the host cell cytosol. Together, the differences among the intracellular bacteria indicate a requirement of host cell lipids that varies among and within vacuolar and cytosolic groups. This review addresses current knowledge on the alteration and use of host lipids during human pathogenic intracellular bacterial infection. The research discussed highlights differences in vacuolar and cytosolic bacteria’s use of host lipids for pathogen membrane structure, host LD alterations during infection, and lipid utilization for energy production.

## 2. Intracellular Bacteria That Replicate Inside a Vacuole during Infection

As briefly mentioned previously, vacuolar bacterial pathogens are defined as species that remain within the vacuole formed upon entry into the host cell, though not all vacuoles are created equally [22,23]. The pathogen-containing vacuoles (PCVs) are very specific to each pathogen with differences in the suitable intravacuolar acidity for pathogen growth. The vacuole markers and composition are indicative of the stages of vacuole transport pathways that the PCV is derived from or halted in and the presence of intravacuolar nutrients and associated host cell organelles. Many vacuolar pathogens, including *Coxiella burnetii*, *Chlamydia* spp., *Mycobacterium* spp., *Anaplasma phagocytophilum*, *Ehrlichia chaffeensis*, *Brucella abortus*, *Legionella pneumophila*, and *Salmonella* spp., have been shown to manipulate host lipids during intracellular infection for an array of defined and speculated functions involved in bacterial and vacuolar membrane production, foam cell presentation and immune modulator production, and energy production. 

### 2.1. Host Lipids Provide Structural Integrity to Pathogen-Associated Membranes

Bacterial membranes are limited in their composition of lipid species when compared to their eukaryotic counterparts. The predominant lipids present in standard Gram-negative and Gram-positive bacterial membranes are cardiolipin, phosphatidylglycerol (PG), phosphatidylethanolamine (PE), phosphatidic acid (PA), and phosphatidylserine (PS), with machinery lacking for biosynthesis of cholesterol and, in some intracellular bacteria, phosphatidylcholine (PC). In general, bacterial membrane lipids are unsaturated and can exhibit distinct branching patterns among the various bacteria [24,25]. Many vacuolar intracellular bacteria recruit and incorporate specific host lipids as structural components for both the host-derived PCVs and bacterial membrane. Although, intracellular bacteria do not encode the enzymes necessary for production of cholesterol and other lipids commonly found within eukaryotic cells, they have adapted mechanisms for modification of sequestered host lipids for incorporation into structural membranes during infection. 

**Figure 1:**
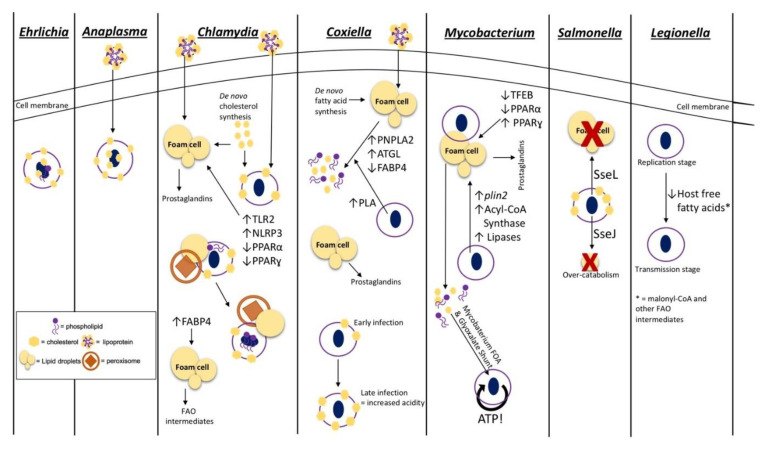
Mechanisms for acquisition and utilization of host lipids by intracellular pathogens. Intracellular pathogens that reside within a vacuole utilize host lipids for various processes during infection. This model depicts the host lipid pathways and methods exploited by various vacuolar bacterial species to enhance intracellular and, ultimately, mammalian infection. The approaches employed by these pathogenic bacteria include (i) lipid acquisition into pathogen-associated membranes as seen in *Ehrlichia*, *Anaplasma*, *Chlamydia*, and *Coxiella* (from left to right); (ii) pathogen- and host-stimulated lipid droplet modulation required during *Chlamydia*, *Coxiella*, *Mycobacterium*, and *Salmonella* infection (from left to right); (iii) immune modulation and lipid inflammatory mediator production from lipid droplets seen during *Chlamydia*, *Coxiella*, and *Mycobacterium* infection (from left to right); (iv) acquirement of host fatty acid β-oxidation (FAO) metabolites for energy production by *Mycobacterium* and modulation of host FAO regulators by *Chlamydia*; (v) regulation of bacterial stage transition of *Legionella* initiated by host intracellular lipid composition. The legend on the bottom left-hand side of the model shows a representative image of the structures used in the panels describing each pathogens’ utilization of host lipids. Toll-like receptor 2 (TLR2); NOD-like receptor protein 3 (NLRP3); peroxisome proliferation activation receptor α (PPARα); peroxisome proliferation activation receptor ɣ (PPARɣ); adipose triglyceride lipase (ATGL); fatty acid binding protein (FABP4); patatin-like phospholipase domain containing protein 2 (PNPLA2); transcription factor EB (TFEB); perilipin 2 (*plin2*).

*Chlamydia* spp. are vacuolar pathogens that typically target epithelial cells in areas of species-specific tropism and can actively replicate in vacuoles within recruited macrophages during infection [26]. A characteristic of the *Chlamydia*-PCV is that the membrane contains Golgi apparatus-derived membrane markers indicative of a non-acidic, nutrient-rich vacuole unlikely to interact with the lysosome during late stages of the endocytic pathway [27,28]. Association of the PCV with the host Golgi apparatus, which acts as a nutrient-rich recycling and trafficking organelle, also provides nutrients to the bacteria. During human and mouse fibroblast infection, the *Chlamydia*-containing vacuole contains cholesterol-rich microdomains that comprise an abundance of PCV membrane-associated bacterial proteins (Figure 1). These bacterial PCV proteins are important for interacting with the host proteins, such as Src-family kinases, to modulate vacuole trafficking via the host microtubule network. This trafficking is required to facilitate the PCV and Golgi-derived endosome fusion for nutrient sharing and genetic exchange required for the development of disease [29,30,31]. In addition to the incorporation of host cholesterol into the PCV, *Chlamydia* spp., such as *C. trachomatis* and *C. pneumoniae*, also actively recruit host cholesterol and free fatty acids through initiation of exogenous lipoprotein recruitment and de novo lipid synthesis for fatty acid overaccumulation within host cells [27,32] (Figure 1). Interestingly, during *C. trachomatis* infection of Hep-2 epithelial cells and mouse and human fibroblasts, host compartments, such as lipid droplets (LDs) and host peroxisomes, along with free fatty acids, colocalize at the PCV [33,34]. The association of these host cellular components at the PCV allows for efficient peroxisome-driven lipolysis of associated fatty acids, specifically PC and lysophosphatidylcholine (lyso-PC), that are unable to be made by *C. trachomatis*. It is important to note that although peroxisome-driven lipolysis is a known source of lipids for the bacteria, lipid droplet-associated lipases are also likely involved in providing the required lipids for *C. pneumoniae* growth [33]. Thus, these events allow *C. trachomatis* to scavenge and modify the catabolized lipids for incorporation in the bacterial membrane [33,35,36,37] (Figure 1). Both host and chlamydial proteins play crucial roles in the sequestering and modification of host PC for incorporation into the bacterial membrane. Specifically, the proteins involved in PC modification are the human proteins, human acyl-CoA:lyso-PC acyltransferase 1, CLA1 lipid transporter, and human acyl-CoA carrier 6, along with the pathogen protein, chlamydial lyso-PC acyltransferase. The combination of these proteins ultimately re-acetylates host PC for bacterial use. These bacterial and host modifications provide PC species with an increased branch number, making these sequestered lipids similar to typical bacteria-made membrane lipids suitable for the production of bacterial progeny [24,38,39,40,41]. *C. trachomatis* also encodes phosphatidylserine decarboxylase and acyl-ACP synthase enzymes supporting the notion that these bacteria have the ability to modify and utilize host lipids during infection [42,43]. Although *C. trachomatis* is able to modify host PCs, this bacteria is also able to produce other phospholipid species utilizing the bacterial type II fatty acid synthesis (FASII) pathway, which when combined with host lipid modification of scavenged lipids, allows for efficient reproduction during intracellular infection [44]. 

*Anaplasma phagocytophilum* and *Ehrlichia chaffeensis* are closely related bacterial species that halt the phagosome–lysosome fusion step of the endocytic pathway and reside within a non-acidic, early and late endosome, respectively, during intracellular infection [45,46]. During mammalian infection, *A. phagocytophilum* targets neutrophils, and *E. chaffeensis* predominantly infects monocytes [46]. Similar to *C. trachomatis*, *A. phagocytophilum* and *E. chaffeensis*, require cholesterol for persistence but do not genetically encode for anabolic cholesterol pathways; thus, cholesterol present within bacterial compartments is captured from the host [47,48]. Similar to *C. trachomatis* PCVs, *A. phagocytophilum* PCVs contain cholesterol-rich domains to enable microtubule trafficking for nutrient-rich endosome fusion, though the specific protein:protein interactions involved have yet to be elucidated [49]. However, unlike *Chlamydia* spp., *A. phagocytophilum* infection of HL-60 promyeloblasts does not require host de novo cholesterol synthesis and, instead, actively stimulates uptake of lipoproteins by the host cell and recruitment to the PCV through regulation of the Niemann–Pick disease type C1 (NPC1)-mediated intracellular cholesterol trafficking [50,51,52]. The mechanism for sequestering cholesterol from the host cell during *E. chaffeensis* infection is not well defined; however, host cholesterol is incorporated into the bacterial membranes of both *A. phagocytophilum* and *E. chaffeensis* (Figure 1). Both *A. phagocytophilum* and *E. chaffeensis* lack the lipid-A protective layer and traditional lipopolysaccharide structures seen in most Gram-negative bacterial outer membranes. Therefore, it is likely that the addition of host cholesterol within the bacterial cell membrane is important in providing rigidity and protection that these bacteria otherwise lack during intracellular infection [50,53]. Recently, *E. chaffeensis* was also shown to encode a partial glycerophospholipid pathway indicating a potential requirement of host glycerophospholipids and intermediates for the production of various membrane lipids. This requirement for host membrane intermediates is supported by the finding that *E. chaffeensis* replication in monocytic THP-1 cells is diminished when host de novo fatty acid synthesis and subsequent phospholipid production is inhibited [49]. The process of sequestering host lipids for *E. chaffeensis* membrane construction is thought to be initiated by a type IV secretion system (T4SS) bacterial effector protein(s), as lipid acquisition is perturbed when bacterial protein synthesis and T4SS secretion is inhibited [49]. 

Conversely, *C. burnetii* promotes phagolysosome maturation, during macrophage infection, to form an acidic parasitophorous vacuole that contains an abundance of host cholesterol and other sterols in the PCV membrane [54]. Though *C. burnetii* infection does not require cholesterol specifically for successful infection, the regulation of the overall sterol composition within the parasitophorous vacuole is necessary for the regulation of infection stages and growth of the PCV [55]. More recent studies indicate that the sterol composition in this PCV is important for regulation of the v-ATPase pump required to maintain optimum acidity within the *Coxiella*-containing vacuole. Specifically, this study showed a correlation of an increase in host cholesterol within the PCV membrane and increases in PCV acidity during later stages of mouse embryonic fibroblast *C. burnetii* infection [56,57] (Figure 1). Inhibition of bacterial protein synthesis followed by a decrease in sterol recruitment suggests that host sterol composition within the PCV is actively regulated by *Coxiella* effector proteins, which likely modulate endosomal trafficking and cholesterol acquisition [58,59]. *C. burnetii* also contains an active sterol reductase, which catalyzes the last step in the cholesterol biosynthesis pathway, implying a possible use of host sterol intermediates for bacterial completion of cholesterol production [60]. Unlike other intracellular pathogens such as *Chlamydia* spp., *E. chaffeensis,* and *A. phagocytophilum*, the use of host cholesterol or lipids for *C. burnetii* membrane production is undefined. The presence of complete bacterial fatty acid and phospholipid biosynthesis pathways within the *C. burnetii* genome suggests that host phospholipid incorporation may not be required for replication. Nevertheless, as described for *C. trachomatis*, host lipid incorporation in bacterial membranes may be coupled with endogenous bacterial membrane production pathways for improved intracellular replication [60]. 

Several studies support the requirement of host lipids for the structural integrity of obligate intracellular pathogens discussed above; however, the requirement of host lipids for de novo membrane synthesis in facultative intracellular bacterial species is not well defined. Genetically, facultative intracellular pathogens contain a higher number of complete biosynthesis pathways overall compared to obligate intracellular bacteria, and the ability to survive and replicate extracellularly indicates a lower reliance on the host for required nutrients [61,62]. However, facultative intracellular *Salmonella* spp. infect macrophages and reside within undeveloped phagosomes that contains host cholesterol thought to be acquired during entry, which plays a role in early vacuolar trafficking [63]. Similar to *Chlamydia*, the cholesterol within the *Salmonella*-PCV is actively recruited to the membrane and is an important regulator of host:pathogen protein interactions by providing structured “hot spots,” such as lipid rafts, on the PCV membrane that facilitate *Salmonella* protein presentation and association with host proteins in the cytosol [64]. This active recruitment is achieved through the type III secretion system (T3SS) effector protein, SseJ, which has acyltransferase activity [65,66,67,68] (Figure 1). A mutation in *sseJ* during mouse infection attenuates virulence and causes a decrease in PCV production, supporting the requirement and use of SseJ as an acyltransferase used to modify membrane lipids for incorporation into the PCV [68].

### 2.2. Host Lipid-Droplet Modulation

Lipid droplets (LDs) are a host cellular compartments used to store predominantly neutral, esterified cholesterol and triacylglycerols, surrounded by a phospholipids monolayer with lipid-associated proteins, which can serve various functions including energy production and immune response modulation [69,70]. Some pathogens, such as *Chlamydia pneumoniae, Chlamydia trachomatis*, *Coxiella burnetii*, and *M. tuberculosis* produce foam cells during macrophage infection, characterized by an overaccumulation of LDs within the host cell [71]. This lipid accumulation in macrophages and the subsequent alteration of metabolism and immune signaling correlate with changes in macrophage polarization, which have been shown to be important during macrophage infection by various intracellular pathogens [72,73].

*C. pneumoniae* and *C. trachomatis* induce a “foamy” cell phenotype during macrophage infection by employing a bacterial cholesterol esterification enzyme, CT149, to form cholesterol esters that are subsequently stored in LDs [42]. During macrophage infection, *C. pneumoniae* also actively downregulates host transcription factors, peroxisome proliferator-activated receptors (PPAR) α and ɣ. These two proteins are both known to be involved in the regulation of LD breakdown and cholesterol efflux, providing a likely mechanism for the formation of the foam cell phenotype during infection [74,75,76,77]. Immune regulation also occurs through the stimulation of the host inflammasome, NLRP3, and toll-like receptor (TLR) 2 to subsequently increase LD formation in infected macrophages. However, the function(s) or consequences of host lipid overaccumulation into LDs during infection by *C. pneumoniae* is not yet clearly elucidated [78,79]. Conversely, *C. trachomatis* employs the modulation of fatty acids within host LDs for immune response regulation by recruiting host triacylglycerol lipases to the LDs associated with the bacterial PCVs. These host lipases breakdown the stored triacylglycerols within the LDs into immune signaling intermediates, such as arachidonic acid, to be converted into immune mediators, such as prostaglandins, for enhancement of tissue damage and overall disease during mammalian infection [40,80]. Stimulation of lipases for prostaglandin production can be induced by pathogen infection of a single cell and by host cell–to–host cell signaling caused by the infection. Therefore, infected and uninfected bystander cells within a mammalian host are likely involved in overall prostaglandin production during infection. As described previously, lipids are also scavenged from PCV-associated LDs for bacterial membrane production [24,38,39,40] (Figure 1). It is important to note that although LDs are a major source of lipids for *C. trachomatis* during intracellular growth, these host structures are not required during mouse embryonic fibroblast infection. Interestingly, during inhibition of LD formation in mouse embryonic fibroblasts, *C. trachomatis* is able to efficiently grow when free fatty acids are supplemented. This suggests that the bacteria are able to recruit free fatty acids and other host lipids not related to LDs to the PCV as a way of compensating for a lack of lipids within LDs [81].

Similarly, *C. burnetii*’s formation of foam cells is actively modulated by bacterial effector proteins and is dependent on an increase in fatty acid recruitment to the endoplasmic reticulum for packaging into host LDs [82]. During *C. burnetii* infection of macrophages, the expression of host proteins involved in LD breakdown, including patatin-like phospholipase domain containing protein 2 (PNPLA2) or adipose triglyceride lipase (ATGL), acyl-CoA:cholesterol transferase, and fatty acid binding protein (FABP4), is modulated suggesting a requirement of host lipids within LDs, such as cholesterol esters and triacylglycerols, during infection [82,83,84,85]. The regulation of ATGL and FABP4 indicate a release of free fatty acids and cholesterol from host LDs for *C. burnetii* nutrient acquisition and membrane production as described previously [82]. *C. burnetii* also encodes a bacterial phospholipase A enzyme that is involved in accumulating free fatty acids, possibly for membrane and energy production or immune regulation during infection [86]. Similar to *C. trachomatis*, *C. burnetii* infection also utilizes prostaglandins derived from host LD intermediates to promote intracellular growth and disease presentation [87,88,89] (Figure 1). Interestingly, the inhibition of acyl-CoA anabolism and de novo lipid synthesis involved in production of host LDs enhances *C. burnetii* growth during macrophage infection. Conversely, pharmacological inhibition of LD catabolism is detrimental to *C. burnetii* infection. Together, these results suggest that lipids derived from host LDs and likely exogenous-derived lipids are essential for modulation of the host immune response and nutrient acquisition during *C. burnetii* macrophage infection [82]. 

Little is known about the mechanisms employed to modulate host LDs during *A. phagocytophilum* and *E. chaffeensis* infection of the respective target cells. During infection of promyelocytic cells, *A. phagocytophilum* increases expression of perilipin 1 (PLIN1), the major host protein important for regulation of LD formation and lipolysis, suggesting a possible role of host LDs as a source of cholesterol and fatty acids. However, the exact function of host LDs for *A. phagocytophilum* infection is still not yet elucidated [90,91,92,93,94]. Interestingly, *E. chaffeensis* has not been shown to have an association with host LDs or LD-related host molecules. Similar to *A. phagocytophilum*, the requirement of host cholesterol for successful infection implies a possible use of host LDs for cholesterol acquisition during infection. 

Facultative intracellular pathogens from the genus *Mycobacterium* provide classic examples of producing a foam cell phenotype required for persistence during alveolar macrophage and overall mammalian infection. Many bacterial and host mechanisms are employed to produce and maintain the foam cell state during mycobacterial infection with various *Mycobacterium* spp. For example, *M. tuberculosis* and *M. leprae* infection increases transcription of bacterial genes involved in LD accumulation, *plin2* (coding for perilipin 2) and acyl-CoA synthase, while *M. tuberculosis* also modulates host transcription factors PPARα and ɣ, similar to *C. pneumoniae* [95,96,97,98,99,100,101]. Unlike *C. pneumoniae*, *M. tuberculosis*’s growth during infection is increased by an upregulation in PPARɣ and subsequent triacylglycerol production and accumulation of host LDs. *M. tuberculosis* growth is also downregulated when LD catabolism is increased by transcription factor EB (TFEB)-initiation and subsequent PPARα expression. Together, these data suggest an importance of LD overaccumulation during macrophage infection [96,97]. Similar to that of *C. pneumoniae* and *C. burnetii*, *M. leprae* infection also increases prostaglandin production, which then stimulates other immune modulatory molecules, such as IL-10 and TGFβ required for bacterial survival and persistence [100,102]. Interestingly, *M. tuberculosis* is capable of producing triacylglycerol-rich bacterial LD storages during the dormancy stage of infection [98,99,103,104,105]; however, *M. tuberculosis* still associates with host LDs suggesting utilization of host storages for nutrient acquisition to increase efficiency and persistence during long-term mammalian infection [106]. *M. tuberculosis* and *M. leprae* also encode phospholipase C and other bacterial lipases used to release free fatty acids from storage lipids as carbon sources for increased infection efficiency [107,108] (Figure 1). Similar to *C. trachomatis*, *M. tuberculosis* is also able to survive in the absence of host LDs suggesting a bacterial mechanism for sequestering free fatty acids from other host sources [109]. 

Host LD accumulation may also be indicative of the virulence potential of various *Mycobacterium* strains. For instance, it is speculated that the formation of LDs is detrimental to less pathogenic strains of *M. tuberculosis* during infection likely due to the increased availability of signaling precursors able to be released from host LDs to produce a bactericidal response [110]. Conversely, infection with the highly pathogenic *M. tuberculosis* strain, H37Rv, supports that increased host LD formation is beneficial for bacterial growth and persistence, in that, the signaling molecules from LDs assist in producing the anti-inflammatory response required for the production of necrotic tissue and granuloma formation during severe mammalian infection [111].

Comparably, *Brucella abortus*, a facultative, intracellular bacteria that resides within the endoplasmic reticulum-associated vacuoles, increases host PPARɣ expression during infection. However, as observed for *M. tuberculosis*, this increase potentially modulates host triacylglycerol production and subsequent accumulation in LDs [112]. Another facultative intracellular pathogen that is thought to associate with host LDs is *Salmonella typhimurium*. Unlike, the pathogens described above, studies have suggested that the association of *S. typhimurium* with host LDs is based on the defined functionality of T3SS effector proteins, such as SseJ and SseL. As mentioned previously, SseJ not only has acyltransferase activity but also possesses cholesterol esterase activity similar to the *C. trachomatis* enzyme used to increase host LD production described above [65,66,67,68]. Infection of host cells with a *C. trachomatis sseJ* mutant results in a decrease in PCV production and in a decrease of LD accumulation. Together, these data support the use of SseJ as a cholesterol esterase for accumulation of stored cholesterol in host LDs during infection [67,68]. On the other hand, SseL contains deubiquitnase activity and *sseL* mutants stimulate an overaccumulation of host LDs (Figure 1). This strongly suggests that SseL and SseJ play a role in the maintenance of lipid homeostasis during infection, for direct bacterial use, and likely play roles in the modulation of the host response as described for other pathogens mentioned [113,114]. 

### 2.3. Fatty Acid β-Oxidation for Energy Production

The ability of bacterial pathogens to generate energy is required for survival and replication during infection of a mammalian host. There are a well-known and characterized range of biosynthesis pathways, both host and bacterial, that have the potential to be employed for energy production. A wide range of bacteria utilize glucose, or a sugar carbon source derivative, as a quick energy source. Glucose is most efficiently used through glycolysis followed by cellular respiration or other aerobic processes [115]. However, glucose and other sugars are not the only carbon sources available and utilized for energy production. Lipids as a carbon source provide more energy per molecule and can be processed through fatty acid β-oxidation (FAO), a pathway found in both microbes and eukaryotes, to produce acetyl-CoA for use in cellular respiration [115,116]. Generally, extracellular bacteria depend less on host processes and encode for the energy pathways necessary for efficient survival, growth, and disease progression. In contrast, intracellular bacteria rely more heavily on nutrients provided by host biosynthesis processes for efficient infection and growth. 

As mentioned previously, facultative intracellular bacteria typically have functional pathways that allow these organisms to survive and replicate in both extracellular and intracellular environments, whereas obligate intracellular pathogens rely heavily on host metabolites during infection [61,62]. For example, *M. tuberculosis* employs multiple mechanisms to sequester host lipids as a carbon source. This facultative intracellular bacteria encodes phospholipases, such as phospholipase C, to breakdown host lipids before employment of bacterial mammalian cell entry (Mce) protein transporter complexes and homologs of flavin adenine dinucleotide (Fad) transporter protein to import cholesterol and fatty acids into the bacterial cell [117,118,119]. Cholesterol and fatty acids are the primary carbon source used for *M. tuberculosis*’s production of energy, making host lipid production important for sustainable and efficient infection [119,120,121,122]. Interestingly, *M. tuberculosis* also encodes for 200 distinct enzymes used for lipid metabolism that are involved in shuttling and processing degraded fatty acids during in vivo infection [107,123,124,125]. There are two main pathways utilized by *M. tuberculosis* for bacterial lipid catabolism, i.e., FAO and the glycoxylate shunt, with the latter being the dominant pathway. Interestingly, the *M. tuberculosis* genome has a large amount of redundancy regarding predicted enzymes within the FAO pathway to predominantly catabolize lipids into acetyl-CoA, which is then shuttled to the tricarboxylic acid cycle (TCA) indicating a potential significance of this secondary pathway during infection [126,127] (Figure 1). The utilization of various lipids as an energy source and the redundancy of lipid metabolic pathways allow *M. tuberculosis* to have enhanced plasticity and adaptability and to persist during long-term mammalian infection [128]. It is important to note that because of the plasticity of *M. tuberculosis* metabolism, cholesterol is not required for active *M. tuberculosis* infection. Nevertheless, the use of cholesterol as a major carbon source is necessary for increased persistence during chronic infections [129]. Although *M. tuberculosis* is able to self-process exogenous cholesterol and lipids as a carbon source through bacterial FAO, a recent study indicates a requirement of active host FAO during infection. Host FAO is important for regulation of antibacterial responses such as reactive oxygen species production and phagosome acidification. *M. tuberculosis*, therefore, is thought to stimulate FAO during infection to promote downregulation of these bactericidal responses [130].

Interestingly, the obligate intracellular bacteria, *Chlamydia* spp. lack a large array of metabolic and lipid biosynthesis pathway thereby relying on the host for pathway intermediates and scavenged lipid nutrients [131]. As described previously, *C. pneumoniae* replication requires the host lipid transport protein, FABP4, which regulates the release of free fatty acids from the host LDs, typically for host FAO and subsequent ATP production. These data suggest that *C. pneumoniae* may utilize host lipids as an intracellular energy source [132] (Figure 1). In addition, a facultative intracellular bacterium, *Legionella pneumophila*, infects alveolar macrophages and replicates within an ER-derived PCV and requires host FAO during infection [133,134,135]. The host FAO regulation has not been shown to be used as a method of energy production for the bacteria but instead is a mechanism for modulating bacterial growth states during the transition from a replicative stage upon host cell entry. This shift in bacterial stages is regulated by nutrient acquisition in the form of ER-derived vacuole fusion with the PCV [136]. Later in the replication stage, modulation of sphingolipid metabolism and autophagy initiates auto-phagolysosome fusion with the PCV providing lysosomal characteristics such as encoded lipolytic enzymes giving potential for breakdown and utilization of other nutrients such as lipids [133,137,138]. Once the PCV is depleted of the nutrients required for active replication, *L. pneumophila* shifts to a “transmission phenotype,” characterized by bacterial flagella activation and a downregulation in bacterial metabolism. During the life stages of intracellular infection, *L. pneumophila* monitors depletion of fatty acid levels, including host malonyl-CoA and intermediates of FAO-derived acyl-chain breakdown, to initiate the stringent response triggered by ppGpp synthetases and SPoT, which regulates the phenotypic shifts required during the *L. pneumophila* lifecycle [134,135,139,140,141,142] (Figure 1).

## 3. Intracellular Bacteria That Replicate within the Cytosol

In contrast to the pathogenic bacteria described above, there is a smaller group of intracellular bacteria that does not actively modulate trafficking of an intracellular vesicular compartment but rather employs mechanisms to escape a membranous vacuole after entry into the host cell to survive and reproduce within the host cytosol. To escape the vacuole, some cytosolic pathogens, including *Listeria monocytogenes* and various pathogenic *Rickettsia* species (spp.), employ bacterial phospholipases and toxins to breakdown the vacuole membrane for exit into the cytoplasm [143,144,145,146,147,148,149,150,151]. Similar to vacuolar pathogens, there is large genomic diversity within the group of cytosolic intracellular bacteria comprised of both facultative and obligate intracellular bacteria [61,62].

### 3.1. Host Lipids Provide Structural Integrity to Bacterial Membranes

The phospholipid and lipid membrane spp. that compose bacterial membranes are similar for most bacterial species as described previously. A distinct difference when discussing membranes associated with vacuolar pathogens and cytosolic pathogens is the presence of a PCV during vacuolar bacterial infection. As discussed previously, various host lipids are recruited to the PCV membrane to provide a variety of functions for the vacuolar bacteria. Cytosolic bacteria, however, do not remain within a vacuole; thus, the incorporation of host lipids into pathogen-associated membranes during replication is limited to the bacterial membrane. Most studies discussing the incorporation of host lipids into the cytosolic bacterial membranes focused on elucidating bacterial membrane compositions and speculated that various lipid classes originated from the infected host cell.

**Figure 2:**
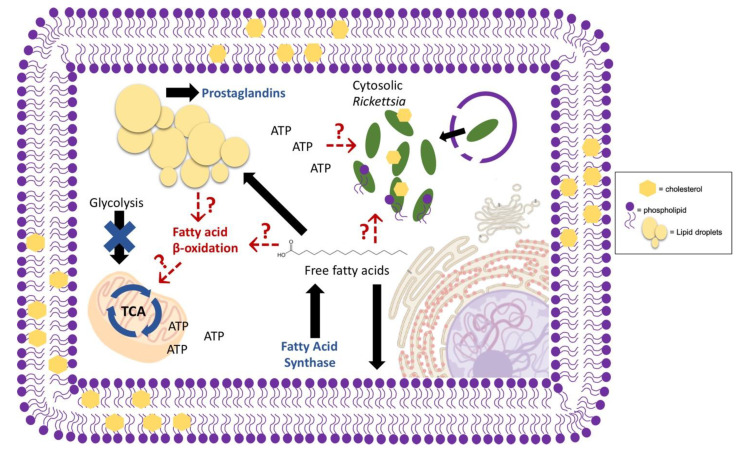
Potential host lipid pathways altered during cytosolic *Rickettsia* infection. A representative cytosolic, intracellular bacteria species, *Rickettsia conorii*, requires host fatty acid synthase, and therefore, free fatty acids for successful replication. Mammalian infection with rickettsial pathogens also alters host prostaglandin production to stimulate the presentation of disease. Metabolically, *Rickettsia* spp. are not self-sufficient and require the host for survival; however, the host pathways required for energy production and subsequent bacterial survival are largely unknown. Evidence suggests that host glycolysis is downregulated leaving other metabolic pathways, such as fatty acid β-oxidation (FAO), as likely candidates for carbon source generation required for oxidative phosphorylation and ATP production. Early studies also suggest the alteration and incorporation of host phosphatidylcholine into the rickettsial membrane, thus indicating a likely requirement of host lipid pathways for bacterial membrane structure during infection. Red arrows indicate speculated host processes and functions of host metabolites during cytosolic infection. Blue figures and words indicate known alterations that occur during infection. Black arrows indicate common pathways and functions of host metabolites. The legend on the right-hand side of the model shows a representative image of the structures use to describe *Rickettsia* utilization of host lipids within infected host cells.

Genomic analysis of various *Rickettsia* spp. and another related bacterial species, *Orientia tsutsugamushi*, indicates gaps within the biosynthetic pathways necessary to construct the bacterial cell wall and membrane structure. This suggests a requirement of host lipid metabolites for membrane production during intracellular replication and growth [152,153,154]. A more recent genomic study denoted multiple lipid anabolic pathways that are missing or incomplete, within various rickettsial genomes, implying a large reliance on the host to provide the enzymes and intermediates necessary for full functional lipid biosynthesis pathways. Among the lipid metabolic pathways lacking or incomplete in the described *Rickettsia* spp. are pathways involved in lipopolysaccharide production and fatty acid and PC synthesis, supporting the implication that there is a heavy rickettsial reliance on host enzymes and metabolic intermediates for lipid products required for adequate building of the bacterial membrane [155]. Interestingly, previous experiments investigating the phospholipid composition of *Rickettsia prowazekii* in chicken embryo yolk sacs indicated the presence of PC and cardiolipins, later found to compose 5.1% and 2.1% of the membrane lipid composition, respectively [156] (Figure 2). Gram-negative bacterial membranes are predominantly composed of saturated and branched fatty acid chains, with limited sterol structures [24,25]. PC is not a normal component of the canonical Gram-negative inner or outer membrane, suggesting that the PC is likely sequestered from the host [157]. Studies have also shown that *Rickettsia rickettsii* membranes contain a moderate amount of unsaturated lipids and that *R. prowazekii* membranes contain cholesterol, in further support that membrane components may be actively acquired from the infected host cell [156,158]. Recently, a proteomic study defining host alterations during *Rickettsia conorii* infection of macrophages showed that inhibition of host fatty acid synthase (FASN), an enzyme required for lipid biosynthesis, is detrimental for bacterial replication and survival. A major role in the host cell and possible requirement of FASN for *R. conorii* growth is the production of phospholipids for membrane production, though the function of host FASN-produced lipids is not defined [159] (Figure 2).

### 3.2. Fatty Acid β-Oxidation for Energy Production

As described for intracellular pathogens that reside inside a vacuole, energy metabolism is an essential factor involved in the maintenance and sustainability of active infection. Due to the genomic decay and increased reliance on host processes and the importance of energy for efficient intracellular pathogen infection, much research has been done to elucidate the host energy pathways required during infection. Although there is an increased reliance on the host metabolism during intracellular infection, some pathogens still contain energy pathways necessary for self-sustainable energy production. One of the major energy-producing pathways is FAO, which catabolizes lipids, either acquired from exogenous lipids, de novo lipids, or lipid storages, for acetyl-CoA generation and use in host or bacterial OXPHOS. It is important to note that when comparing energy metabolism between vacuolar and cytosolic intracellular pathogens, the nutrients within the cellular compartments are vastly different. For example, vacuolar bacteria have adapted to utilize a diverse range of metabolites that are recruited and associated with PCVs during infection. In contrast, cytosolic bacteria do not employ this strategy and are limited to using available metabolites within the cytosol for energy production. 

A study of host metabolites present during *L. monocytogenes* infection of *Drosophila melanogaster*, for example, showed an overall downregulation in energy metabolites, such as triglycerides, glycogen storages, and other intermediates required for the major energy pathways, FAO and glycolysis. This downregulated shift in metabolism is speculated to be an antibacterial response to prevent bacterial use of energy stores or to be a bacterial mechanism employed to block antibacterial responses, such as use of major energy metabolites for regulation of reactive oxygen species used by the host cell as an antibacterial mechanism, as seen with *M. tuberculosis* [130,160]. 

Similar to *Mycobacterium* spp. variation in virulent potential, *Rickettsia* spp. also vary in their capacity to cause disease in mammals. A recent study demonstrated that a recognized human pathogen, *R. conorii*, is able to invade into and efficiently replicate within both non-phagocytic and phagocytic mammalian cells. In contrast, *Rickettsia montanensis*, a species that is not recognized as pathogenic to mammals, can proliferate in epithelial cells but is rapidly killed intracellularly in macrophage-like THP-1 cells. This study suggested that growth within professional phagocytic cells may be a key determinant to distinguish pathogenic *Rickettsia* spp. from non-pathogenic species [161]. In addition, a proteomic study revealed the differential host protein changes that occur when macrophage-like THP-1 cells are infected with either *R. conorii* or *R. montanensis*. In the *R. conorii* infected cells, host glycolysis and other sugar metabolic processes were downregulated while proteins involved in OXPHOS were upregulated. In contrast, infection with *R. montanensis* did not result in these changes. The observed overall increase in OXPHOS and decrease in glycolysis during *R. conorii* infection of macrophages, strongly suggested that non-sugar carbon sources, such as lipids, are likely important for energy production. This proteomic analysis also indicated a shift in host lipid metabolic pathways and a requirement of the anabolic FASN, discussed previously, for de novo production of host lipids (Figure 2). These metabolic shifts are also not observed during *R. montanensis* infection of macrophages. Taken together, these results suggest that the ability to stimulate specific host metabolic pathways within macrophages and possibly other target cells likely contributes to the virulence potential of *R. conorii* and related pathogenic spotted fever group *Rickettsia* species [159]. Similar to *R. conorii*, the closely related cytosolic bacteria *O. tsutsugamushi* also shifts the host cell metabolic response to decrease host glycolysis during in vivo infection of a mouse model, suggesting a likely use of lipids as carbon sources. However, the *O. tsutsugamushi* preferred carbon source for energy production during infection is still unclear [162]. Although the use of the newly synthesized lipid species is yet to be defined, it is possible that these fatty acids could be used as a carbon source for ATP production during rickettsial macrophage infection [159]. In support of this hypothesis, a previous study of another human pathogenic species, *Rickettsia typhi*, identified a gene, *tlc1*, present in rickettsial genomes that encodes active transporter that is sufficient for ATP transport [163]. Similarly, the *O. tsutsugamushi* genome also contains genes predicted to encode redundant ATP transport systems and lacks certain enzymes necessary for ATP production [164]. Together, these data strongly suggest that this class of obligate, intracellular bacteria acquire host-derived ATP to meet metabolic requirements. 

Another pathogen that manipulates the host during infection for regulation of host energy production is the facultative intracellular bacteria, *Francisella tularensis*. Similar to *Rickettsia*, studies have suggested that *F. tularensis* initiates utilization and acquisition of alternative carbon sources, such as glutamate and fatty acids, during infection. These sequestered metabolites are used for mitochondrial respiration and host ATP production during early stages of infection. Although the host metabolic profile is known, the exact mechanism and advantage to the host and/or bacteria has yet to be defined [165].

### 3.3. Host Lipid-Droplet Modulation

Unlike some of the vacuolar pathogens, the foam cell phenotype is not defined among cytosolic pathogens. *O. tsutsugamushi* stimulates an increase in triacylglycerol-rich LDs in murine fibroblasts suggesting that these LDs may serve as a nutrient source for energy production [166]. Conversely, infection of *Drosophila melanogaster* with *L. monocytogenes*, results in a decrease in triacylglycerols and subsequent LD structures during infection in this model system, suggesting lipids within host LDs are likely being utilized during infection for functions such as host energy production discussed previously [160]. In contrast, rickettsial species have not been associated with the utilization of host LDs for energy production; however, these bacteria do encode phospholipases within the genome. Phospholipases are enzymes that have been linked with other bacterial species described previously for generation of LD-derived lipid immune regulators. Specifically, the phospholipase A_2_ enzymes have been shown to be present and functional in *R. typhi* and *R. prowazekii* suggesting the ability to actively release fatty acids from host LDs for functions yet to be elucidated [150,151,167,168,169]. Among the *R. prowazekii* and *R. typhi* phospholipase enzymes are RP534 and RT0522, respectively, that are proteins homologous to *Pseudomonas aeruginosa*, ExoU, which hydrolyzes fatty acids from LD for the production of prostaglandins. Some studies have demonstrated that *Rickettsia* spp. stimulate an increase in prostaglandin production during in vivo and in vitro infection models indicating that these mechanisms discussed could be used for the production of lipid immune modulators derived from host LDs as described for *C. pneumoniae*, *C. burnetii*, and *M. leprae* [170,171,172] (Figure 2). Similarly, *L. monocytogenes* and *Shigella dysenteriae* also increase prostaglandin levels during in vivo infection of a mouse model for immune suppression, suggesting LD release of lipid modulators, however, the mechanism has yet to be defined [173,174]. In addition, *F. tularensis*, a facultative intracellular bacteria that resides within the late phagosome prior to release into the cytosol, modulates host TLR2 signaling and the transcription factor PPARα, which as described for *C. pneumoniae* can have implications on LD production during macrophage infection [175,176,177,178]. Moreover, similar to other cytosolic bacterial infections, *F. tularensis* macrophage infection also increases host prostaglandin production via host phospholipases [178,179,180]; although, bacterial factors directly involved in prostaglandin release and other LD functions during *F. tularensis* infection are still unknown. 

## 4. Conclusions

Both obligate and facultative intracellular bacteria have developed several strategies to regulate and manipulate host processes for efficient intracellular growth. Among these is the modulation of host lipid metabolism that has been demonstrated not only to be required for the maintenance of host cell homeostasis but also to be implemented in various intracellular infections for increased persistence and disease progression. The key lipid pathways employed by intracellular bacterial pathogens for proliferation and sustainable infection include, but are not limited to, membrane fatty acid biosynthesis, LD regulation, and lipid catabolism through FAO for energy production. The current knowledge on host lipid metabolism utilization by various species of intracellular bacteria is fairly limited, in that, much of the previous research addresses the recruitment and sequestering of lipids to the PCV for various functions during infection with bacteria that reside within a vacuole. As demonstrated here, there are large experimental gaps, specifically in facultative and cytosolic intracellular bacteria, where host lipids have the potential to play specific roles in infection; however, the functions and mechanisms involved in this use of host lipid metabolic processes are not well defined compared to some of their vacuolar counterparts. Therefore, it is necessary to further investigate the importance of host lipids for intracellular bacterial infection to better understand the host:pathogen relationship required to provide potential targets for increases in therapeutics against these human disease causing agents.
